# Synthesis and biological evaluation of coumarin-quinone hybrids as multifunctional bioactive agents

**DOI:** 10.5599/admet.1468

**Published:** 2022-10-07

**Authors:** Anees Pangal, Khursheed Ahmed

**Affiliations:** 1 Department of Chemistry & Post Graduate Centre, Abeda Inamdar Sr. College of Arts, Science & Commerce (Autonomous), Camp, Pune – 411001, India; 2 Advanced Scientific Research Laboratory, Azam Campus, Pune – 411001, India

**Keywords:** ADMET, Molecular Docking, Antiproliferative, Antimicrobial, Antioxidant, drug-likeness

## Abstract

We report the synthesis, structural characterization and pharmaceutical activity of four coumarin-quinone hybrids. The compounds were significantly active against *Staphylococcus aureus*, *Pseudomonas aeoginosa* and *Candida albicans*. Promising antioxidant activity was observed when compared to ascorbic acid. Two compounds, DTBSB and DTBSN, also showed commendable *in vitro* antiproliferative activities against the cells of human cancer cell lines MCF-7, MDA-MB-231, COLO-205, HT-29 and A549 along with appreciable tumor selectivity with distinct selectivity index. Molecular docking studies using cyclooxygenase-2 (PDB ID: 6COX) revealed strong binding affinities for the COX-2 active site. Moreover, ADMET properties of the synthesized compounds were determined using the pKCSM and SwissADME online tools and all the compounds had accurate pharmacokinetic profiles. Hence, the new coumarin-quinone hybrids DTBSB and DTBSN can be considered for optimization and lead development.

## Introduction

Natural and synthetic coumarins have gathered much attention based on a broad spectrum of pharmacological and biological properties. In the literature, coumarin and its derivatives exhibit comprehensive biological activities [1] like anticancer [2-5], antioxidant and anti-inflammatory [6], antimicrobial [7], antifungal [8] and anti-HIV [9] activities. Natural and synthetic coumarin compounds like esculetin and scopoletin have potential as anticancer agents. Naturally, coumarin displayed antiproliferative effects on various types of cancers such as prostate, renal, breast, laryngeal, lung, colon, CNS, leukemia and melanoma [10-11]. Coumarins are used in the treatment of different types of cancers like prostate cancer, renal cell carcinoma and leukemia and to inhibit the spread of tumors [12]. The wide scope of biological activities attributed to coumarins and their medicinal impact on various disorders have received considerable attention. Furthermore, the stability and solubility of coumarins are intriguing, which are important criteria for drug development [13].

Natural quinones such as juglone and plumbagin exhibited growth inhibitory effects on microorganisms [14]. Benzoquinones play an important role in oxidative phosphorylation electron transfer processes and bioenergetic transport [15]. Quinones showed diverse pharmacological properties such as anti-inflammatory [16], antimicrobial [17] and anticancer [18-20]. 1,4-benzoquinone derivatives were applied as antibiotics [21-22], antitumor [23-25], antimalarial [26] and anticoagulant compounds [27]. The anticancer properties of quinones are well described and there are several clinically important antitumor drugs based on quinone scaffolds, e.g., anthracyclines, mitoxantrones and saintopin [28].

Cheminformatic tools, including the Prediction of Activity Spectra for Substances (PASS), Lipinski’s rule of five, and ADMET (absorption, distribution, metabolism, and excretion – toxicity) predictions, are useful applications for the optimization of drug discovery [29]. ADMET, which constitutes the pharmacokinetic profile of a drug molecule, is essential for the evaluation of its pharmacodynamic properties [30]. ADMET Predictor is a program designed for the estimation of pharmacokinetic parameters or properties based on molecular structures of drug candidates [31]. The ideal oral drug is rapidly and completely absorbed by the gastrointestinal tract, is distributed specifically to its site of action in the body, is metabolized in a way that does not instantly diminish its activity, and is eliminated without causing harm. Therefore, ADMET properties are important determinants of the test compound for any future therapeutic application in humans [32].

In this work, we combined coumarin and quinone moieties to obtain new hybrid molecules with promising biological activities such as antimicrobial, antioxidant and anticancer properties. The interaction and binding affinities of the new test compounds with cyclooxygenase (COX) were determined via docking calculations. The drug-like potential of these hybrids was evaluated using *in silico* methods. Of the four synthesized coumarin-quinone hybrids, DTBSB and DTBSN were found to be more effective biological agents among the synthesized analogs.

## Experimental

### Materials

Solvents were purchased from commercial sources and were dried by standard protocols. The starting materials such as ethyl 3-hydrazinyl-3-oxopropanoate, 2,6-di-*tert*-butyl-1,4-benzoquinone, 5-bromosalicylaldehyde, 5-chlorosalicylaldehyde, and 5-nitrosalicylaldehyde were obtained from Sigma-Aldrich, India. Salicylaldehyde, trifluoroacetic acid, piperidine, acetone, methanol, ethanol and dichloromethane were obtained from SD-FCL Chemical Limited, Mumbai, India. DMEM and FBS were purchased from Himedia, Mumbai. The MTT (3-(4,5-dimethyl-2-thiazolyl)-2,5-diphenyl-2*H*-tetrazolium bromide) reagent was obtained from G-Biosciences, USA. TLC was monitored using commercially available aluminium TLC plates coated with silica gel GF254, and the developed plates were visualized by UV light and iodine vapors. Melting points of synthesized compounds were determined with an open capillary tube using a VEEGO melting point apparatus. The HRMS, FTIR and NMR spectroscopic data were obtained from CIF, Savitribai Phule Pune University, Pune and CIF, IISc, Bangalore. The *in vitro* biological activities and *in silico* predictions were carried out at Advanced Scientific Research Laboratory, Abeda Inamdar Senior College, Pune.

### Synthesis of coumarin-quinone hybrids

**Figure 1.**
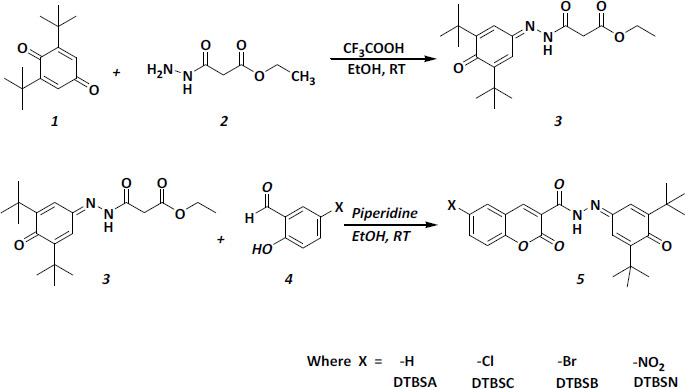
Synthesis of coumarin-quinone hybrids (**5**)

The procedure used for the synthesis of target coumarin-quinone hybrids compounds is illustrated in Figure 1. 2,6-di-*tert*-butyl-1,4-benzoquinone (**1**, 10 mmol) and ethyl 3-hydrazinyl-3-oxopropanoate (**2**, 10 mmol) was dissolved in ethanol. After complete dissolution, a few drops of trifluoroacetic acid were added and the mixture was refluxed for 2 hours. The reaction mixture was poured onto an ice/water mixture and the separated product was collected, washed with cold water several times, and recrystallized from ethanol to obtain 2,6-di-*tert*-butyl-1,4-benzoquinone hydrazone (**3**).

A mixture of (**3**) and substituted salicylaldehyde (**4**, 1eq) was dissolved in ethanol. A few drops of piperidine were added to the mixture, and the reaction mixture was stirred at room temperature until the reaction was completed, which was monitored by TLC. After completion of the reaction, the reaction mixture was neutralized with dil. HCl and formed coumarin-quinone hybrids (**5**) were isolated by filtration and recrystallized in ethanol.

The detailed physical properties and spectral characterization data are given below.

Ethyl 2-(3,5-di-tert-butyl-4-oxocyclohexa-2,5-dienylideneaminocarbamoyl)acetate (**DTBH**): Yellow solid (95 %), MP 164-166 °C, HRMS (EI): C_19_H_29_N_2_O_4_, [M+H] = 349.21, FTIR(cm^-1^): 3739.30 (N-H), 1744.30 (lactone C=O), 1683.55 (C=O), 1624.73 (C=O), 1536.02 (C=N), 1315.21 (C=C), 1141.65-1024.98 (C-O),, ^1^H-NMR (400MHz, *d-DMSO*, (δ,ppm): 1.23 (s, 18H), 4.10 (q, 2H), 3.74 (s, 2H), 1.21 (t, 3H), 6.82 d, 1H), 7.74 (d, 1H), 12.39 (s, 1H, –NH), ^13^C-NMR (125MHz, *d-DMSO*): 186.77, 166.03, 158.42, 154.57, 151.10, 150.61, 146.68, 135.22, 133.21, 130.26, 125.94, 118.52, 117.20, 116.13, 35.55, 29.46

N'-(3,5-di-tert-butyl-4-oxocyclohexa-2,5-dienylidene)-2-oxo-2H-chromene-3-carbohydrazide (**DTBSA**): Light orange solid (85 %), MP 134-136 °C, HRMS (EI): C_24_H_27_N_2_O_4_, [M+H] = 407.20, FTIR(cm^-1^): 3739.30 (N-H), 1712.48 (lactone C=O), 1683.15 (C=O), 1624.22 (C=O), 1525.42 (C=N), 1301.72 (Aromatic C=C),1202.40-1024.98 (C-O), ^1^H-NMR (400MHz, *CDCl_3_*) (δ, ppm,): 1.34 (s, 18H), 12.78 (s, 1H- NH), 9.15 (s, 1H), 7.40 to 7.79 (m, 4H), 8.5 (d, 1H), 7.04 (s, 1H), 6.98 (s, 1H), ^13^C-NMR (125MHz, *CDCl_3_*): 186.77, 166.03, 158.42, 154.57, 151.10, 150.61, 146.68, 135.22, 133.21, 130.26, 125.94, 118.52, 117.20, 116.13, 35.55, 29.46.

N'-(3,5-di-tert-butyl-4-oxocyclohexa-2,5-dienylidene)-6-chloro-2-oxo-2H-chromene-3-carbohydrazide (**DTBSC**): Orange solid (90 %), MP 186-188 °C, HRMS(EI): C_24_H_26_ClN_2_O_4_, [M+H] = 441.16, FTIR (cm^-1^): 3739.30 (N-H), 1712.48 (lactone C=O), 1525.42 (C=N), 1301.72 (Aromatic C=C), 1202.40-1024.98 (C-O), ^1^H-NMR (400MHz, *CDCl_3_*) (δ, ppm,): 1.30 (s, 18H), 12.67 (s, 1H, -NH), 9.07 (s, 1H), 7.75 (d, J=7.69Hz, 1H), 7.69 (dd, J=7.69 and 8.8 Hz, 1H), 7.42 (d, J=8.8Hz, 1H), 7.02 (s, 1H), 6.96 (s, 1H), ^13^C-NMR (125MHz, *CDCl_3_*): 186.68, 167.08, 157.93, 154.41, 153.91, 150.52, 149.29, 146.97, 135.07, 133.29, 129.12, 118.37, 116.09, 115.62, 35.47, 29.44.

N'-(3,5-di-tert-butyl-4-oxocyclohexa-2,5-dienylidene)-6-bromo-2-oxo-2H-chromene-3-carbohydrazide (**DTBSB**): Brown solid (92 %), MP 152-156 °C, HRMS(EI): C_24_H_26_BrN_2_O_4_, [M+H] = 485.11, FTIR (cm^-1^):3745.08(N-H), 1712.48 (C=O), 1640.16 (C=O), 1525.10 (C=N) 1363.43 (C=C), 1197.58-1014.37 (C-O), ^1^H-NMR (400MHz, *CDCl_3_*) (δ, ppm,): 1.34 (s, 18H), 12.67 (s, 1H, -NH), 9.061 (s, 1H), 7.91 (s, 1H), 7.83 (d, J=8.8 Hz, 1H), 7.36 (d, J=8.8 Hz, 1H), 7.17 (s, 1H), 6.86 (s, 1H), ^13^C-NMR (125MHz, *CDCl_3_*): 186.71, 167.08, 161.55, 157.91, 154.41, 150.50, 149.18, 146.96, 137.85, 133.09, 132.76, 118.65, 116.36, 115.64, 36.38, 29.45.

N'-(3,5-di-tert-butyl-4-oxocyclohexa-2,5-dienylidene)-6-nitro-2-oxo-2H-chromene-3-carbohydrazide (**DTBSN**): Dark brown solid (92 %), MP 118-120 °C, HRMS(EI): C_24_H_26_N_3_O_6_, [M+H] = 452.18, FTIR (cm^-1^):3739.30(N-H), 1681.62 (C=O), 1337.39 to 1509.99 (Aromatic, -NO_2_), 1293.04-1020.16(C-O), ^1^H-NMR (400MHz, *CDCl3*) (δ, ppm,): 1.28 (s, 18H), 10.00 (s, 1H, -NH), 9.21 (s, 1H), 8.70 (d, J=2.4Hz, 1H), 8.07 (dd, J=2.4 and 2.8 Hz, 1H), 7.91 (d, J=2.8 Hz, 1H), 7.26 (s, 1H), 6.84 (s, 1H), ^13^C-NMR (125MHz, *CDCl_3_*): 186.74, 165.42, 159.32, 153.62, 151.61, 149.17, 144.96, 138.61, 136.54, 132.96, 128.04, 118.31, 116.85, 115.80, 34.38, 29.51.

### Antimicrobial activity

The nutrient broth was prepared and autoclaved for 20 minutes at 120 psi. Cultures of *Staphylococcus aureus* NCIM 5021 and *Pseudomonas aeruginosa* NCIM 5029 were inoculated and incubated at 37 °C for 24 hours. Then, each well was inoculated with a microbial inoculum prepared in the same medium after dilution of standardized microbial suspension adjusted to 0.5 McFarland scale (10^8^ CFU/mL). 180 μl of the cell suspension was seeded in each of the 96 well plates and 20 μl of different concentrations of the test compounds and the standard was added. The wells without test compound were considered as control and streptomycin was used as standard. After mixing, the 96-well plates were incubated at 37 °C for 24 hours. After incubation, the absorbance of each well was recorded at 620 nm using Readwell Touch Automatic Elisa Plate Reader (Robonik India Private Limited). All the experiments were performed in triplicates and the growth percentage was calculated using the formula:



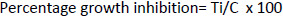



where Ti = Growth of the microorganisms in the presence of a drug, and C = Control growth.

A similar procedure was applied for the antifungal activity test with slight changes. Potato dextrose broth (PDB) was used for fungal culture pre-enrichment, dilutions and inoculations. The antifungal activity was evaluated using the *Candida albicans* NCIM 3100 strain. 180 μl of the cell suspension was seeded in each of the 96-well plates and 20 μl of different concentrations of the test compounds and the standard was added to the respective wells in the plate. The wells without test compound were considered as control and itraconazole was used as standard. After mixing, the 96-well plates were incubated at 37 °C for 24 hours. After incubation, the absorbance of each well was recorded at 620 nm using Readwell Touch Automatic Elisa Plate Reader (Robonik India Private Limited). All the experiments were performed in triplicates and the growth percentage was calculated using the formula mentioned above.

### Antioxidant activity (DPPH scavenging activity)

The DPPH free radicals scavenging activity was assessed using the microplate assay standard method with slight modifications. Drug stock solutions (1000 μg/ml) were diluted to final concentrations of 1, 5, 10, 20, 25, 40, 50, 60, 80, 100, 125 and 150 μg/ml in methanol. 0.004 g DPPH reagent was dissolved in 100 ml methanol. 50 μl of the sample and 150 μl of the DPPH solution were added to 96 well plates. The plate was incubated for 30 min at room temperature in the dark. The absorbance (Abs) was recorded at 520 nm wavelength with a microplate reader (Readwell Touch Automatic Elisa Plate Reader (Robonik India Private Limited) and converted to the percentages. Methanol was used as a solvent and ascorbic acid as standard. The percentage radical scavenging was calculated from the absorbance using the following formula:



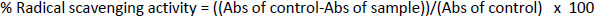



### Cell viability inhibition assay (MTT Assay)

The *in vitro* antiproliferative activities of the test compounds were evaluated against five human cancer cell lines, which include two human breast cancer cell lines (MCF-7 and MDA-MB-231), two human colon carcinoma cell lines (COLO-205 and HT-29), and one human lung carcinoma (A549) using the MTT assay. 5-Fluorouracil (5-FU) was used as a positive control. The tested cell lines were purchased from The National Centre for Cell Science (NCCS), Pune, and cultivated in the appropriate growth medium. The growth medium was supplemented with 100 mg/mL of streptomycin, 100 units/mL of penicillin, and 10 % of heat-inactivated fetal bovine serum in a humidified 5 % (v/v) CO_2_ atmosphere at 37 °C. Then 1x10^6^ cells per well were seeded into 96-well well plates. The medium was replaced after 24-48 hours with a fresh medium containing different dilutions of the test compounds (diluted using the DMEM). After 48 hours, 5 % MTT solution was added. After incubation for additional 4 hours, the formazan formed by metabolically viable cells was dissolved in DMSO, and after 10-20 minutes, absorbance was recorded at 570 nm using Readwell Touch Automatic Elisa Plate Reader (Robonik India Private Limited). The metabolic viability of cancer cells treated with test compounds was compared with the viability of untreated cells (taken as 100% viable). All experiments were performed in triplicates and the growth percentage was calculated using the formula:



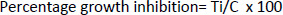



where, Ti = Growth of the microorganisms in the presence of test compound and C = Control growth.

### In vitro cytotoxicity assay against non-cancerous cells

To study the toxicity against non-malignant cells, the compounds were tested against non-cancerous normal human peripheral blood mononuclear cells (PBMCs). Isolation of peripheral blood mononuclear cells was done using Ficoll-Hypaque according to the standard method [33] and an MTT assay was used to evaluate the cytotoxicity of the synthesized hydrazones. 1×10^6^ cells per well were seeded in a 96-well plate before they were exposed to different concentrations of tested compounds for 48 hrs along with a control well. After 48 hrs, the culture medium containing hydrazones was removed by washing with PBS. Then 10 μl of MTT solution (5mg/ml) was added and incubated for 4 hours, followed by the addition of 100 μl of DMSO. After 10-20 minutes, absorbance was recorded at 570 nm using Readwell Touch Automatic Elisa Plate Reader (Robonik India Private Limited).

Furthermore, the selectivity index was calculated by using the following formula.

Selectivity index = IC_50_ value from PBMC/IC_50_ value of the same compound in cancer cells

### Molecular docking

Molecular docking studies were carried out with the COX-2 protein model [34]. Essential hydrogen atoms, Kollman united atom type charges and salvation parameters were added with the aid of AutoDock 4.2 tools. Molecular docking was conducted in these protein cavities by using Autodock Vina. Auto Dock 4.2 (MGL tools-1.5.6) was used to perform all docking calculations, and finally, Pymol viewer was used to visualize docking results.

### ADMET and pharmacokinetic studies

ADMET and pharmacokinetic properties were determined using pkCSM (A Cambridge online source, link: http://biosig.unimelb.edu.au/pkcsm/prediction). The structures of all test compounds and their physicochemical properties were drawn and calculated using ChemDraw 12.0 software. Simultaneously, the SMILE file format of all compounds was obtained from ChemDraw 12.0 to obtain the drug-likeness data. pkCSM predictor provides information regarding absorption parameters like human intestinal absorption (HIA), oral bioavailability, Caco-2 permeability, distribution parameters like plasma protein binding (PPB), blood-brain barrier (BBB), metabolism parameters like cytochrome P450 2D6 (CYP2D6) inhibition and cytochrome P450 3A4 (CYP3A4) inhibition, excretion parameters like renal clearance, and toxicity parameters like organ toxicity and genomic toxicities. The pharmacological properties and drug-likeness of the test compounds were evaluated using the online source SwissADME (link: http://www.swissadme.ch/).

## Results and discussion

### Chemistry

The target compounds were synthesized in two steps, as shown in Figure 1. Initially, 2,6-di-*tert*-butyl-1,4-benzoquinone hydrazone (**3**) was prepared from the reaction of 2,6-di-*tert*-butyl-1,4-benzoquinone (**1**) with ethyl 3-hydrazinyl-3-oxopropanoate (**2**). The intermediate (**3**) was obtained as a yellow compound in high yield. The structure of (**3**) was confirmed based on its HRMS, IR, ^1^H-NMR and ^13^C-NMR spectra.

Subsequently, the coumarin-quinone hybrids (**5**) were obtained from (**3**) and the corresponding salicylaldehyde (**4**) in the presence of a few drops of piperidine, followed by neutralization with diluted HCl. Four coumarin-quinone hybrids abbreviated as DTBSA, DTBSC, DTBSB and DTBSN were obtained and purified, followed by characterization using HRMS, FTIR, ^1^H-NMR and ^13^C-NMR spectroscopy.

The HRMS (EI) spectra of all coumarin-quinone hybrids showed the major peaks corresponding to the expected M+1 or M+H fragment at 407.20, 441.16, 485.11 and 452.18, respectively. The IR spectra of these analogs showed characteristic peaks for –NH, lactone carbonyl and >C=O (amide). The IR spectrum of all analogs showed one peak between 3739.30 to 3745.08 cm^-1^ for –NH. The peaks at 1712 to 1714 cm^-1^ is due to >C=O (lactone moiety) and at 1624.73 to 1683.55 cm^-1^ are amide >C=O. The peak for the imine group was detected between 1525 to 1536 cm^-1^. The ^1^H-NMR spectra of the hybrid compounds showed the olefin proton of the coumarin ring as a sharp singlet at 9.06 to 9.5 ppm. The amide protons appeared as singlet from 10 to 12.78 ppm. The ^13^C-NMR of all these analogs exhibited signals for all aromatic carbon atoms in the estimated range and characteristic peaks of imine, lactone carbonyl, amide and ketone carbonyl carbons in the range of 153-154, 157-159, 165-167 and 186 ppm, respectively.

### Antimicrobial activity

**Figure 2.**
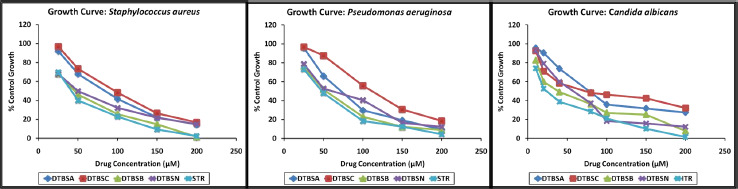
Results of antimicrobial activity

The coumarin-quinone hybrids (**5**) were tested for their antimicrobial activity using *Staphylococcus aureus* NCIM 5021, *Pseudomonas aeruginosa* NCIM 5029 and *Candida albicans* NCIM 3100 strains. Different concentrations of the hybrids were used for screening. The obtained results were compared with the values produced from the standard drugs streptomycin (antibacterial) and itraconazole (antifungal). Growth curves are shown in Figure 2. Each compound showed plausible antimicrobial activity in a dose-dependent way. Among the synthesized derivatives, DTBSB was most active against *Staphylococcus aureus*, followed by DTBSN, DTBSA and DTBSC. DTBSB was most active against Gram-negative organism *Pseudomonas aeruginosa* by 90 % at the highest concentration. DTBSC was found to be the least active analog, with about 20 % bacterial growth at higher concentrations. The screening data indicated that DTBSB showed significant activity against *Candida albicans.*

IC_50_ was calculated and tabulated in Table 1. In terms of the IC_50_ values, all the analogs have good to moderate antimicrobial activity against the three microorganisms. The standard compounds streptomycin (STR) and itraconazole (ITR) exhibited the IC_50_ value of 76.96 ± 4.46 μM and 56.49 ± 0.82 μM, respectively. Compared to STR, DTBSB exhibited good antibacterial activity against Gram-positive bacteria *Staphylococcus aureus* (IC_50_ = 107.28 ± 1.23 μM). In terms of the IC_50_ value, the analogs DTBSB was also most active against *Pseudomonas aeruginosa* (IC_50_ = 129.63 ± 2.50 μM), while DTBSC was the least active analog. The IC_50_ value of DTBSB was close to the IC_50_ value of the control compound STR. When compared with STR, the synthesized analogs exhibited good to moderate antibacterial potential against Gram-positive bacteria *Pseudomonas aeruginosa*. Further, all analogs possessed moderate antifungal activity against *Candida albicans*. Among the synthesized compounds, the most potent analog is DTBSB and exhibited an IC_50_ value of 121.04 ± 1.10 μM. The IC_50_ value of standard ITR was 56.49 ± 0.82 μM. The other derivative expressed higher IC_50_ values, which makes them less susceptible to *Candida albicans*. When compared with ITR, the new analogs have moderate antifungal potential.

The most active derivative, DTBSB, can be selected for further development and optimization as an antimicrobial agent can be selected for further development and optimization as an antimicrobial agent.

### Antioxidant activity

**Figure 3.**
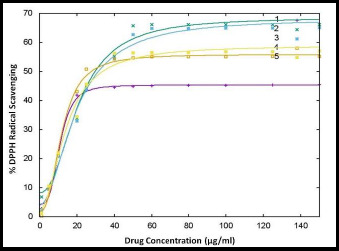
Results of antioxidant activity (1=STD, 2=DTBSA, 3=DTBSC, 4=DTBSB, 5=DTBSN)

All coumarin-quinone hybrids were evaluated for their antioxidant activity using the DPPH assay. The obtained results after drug treatment are depicted in Figure 3.

The scavenging activity (in %) versus the concentration of test compounds was plotted and presented in Figure 3. The IC_50_ concentration values were determined from an online source (http://www.IC50.tk). The results obtained were compared with the IC_50_ value of ascorbic acid (Standard/STD). The results revealed that DTBSB and DTBSN were highly active. The other analogs showed good antioxidant activity when compared with the standard. The scavenging effect of the test compounds increased in a dose-dependent manner. The IC_50_ values for the hybrids were 10.82, 19.20, 18.71, 11.63 and 13.90 μg/ml, respectively, which were comparable to the IC_50_ value of ascorbic acid. Among the new hybrid compounds, DTBSN exhibited the best antioxidant activity.

### Anticancer studies

**Figure 4.**
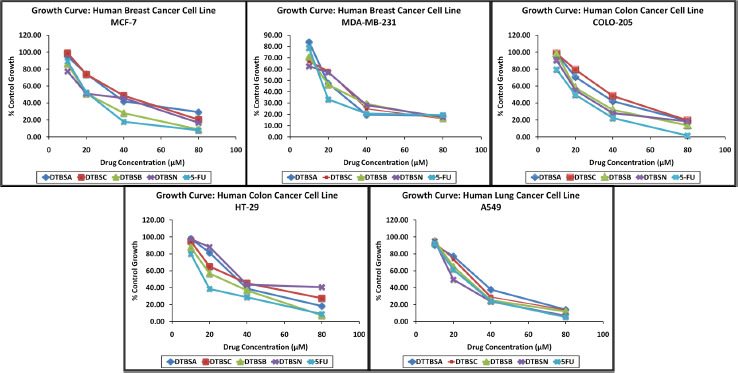
Effect of coumarin-quinone hybrids concentration on cancer cell growth at the indicated doses, μM

All coumarin-quinone hybrids were evaluated for their *in vitro* antiproliferative activity against a set of human cancer cell lines, which includes two breast cancer cell lines (MCF-7 and MDA-MB-231), two colon carcinoma cell lines (COLO-205 and HT-29) and one lung carcinoma (A549). All experiments were performed in triplicates and the obtained results are expressed as the percent growth (%) at different concentrations of test compounds expressed in μM. 5-Fluorouracil (5-FU) was used as standard. Drug concentration causing 50 % inhibition of cell growth was characterized as IC_50_ value. The results revealed that all coumarin-quinone hybrid compounds exhibited considerable activity against all tested cancer cell lines. Graphs of the dose-dependent effects of the hybrids on these cell lines are shown in Figure 4.

From the antiproliferative screening data, it is apparent that with an increase in the concentrations of the tested compounds, the antiproliferative potential of the hybrids increases, too. The IC_50_ values were calculated using the graph in Figure 4. The results are shown in Table 2. The hybrids DTBSB and DTBSN exhibited good antiproliferative activity against all cancer cell lines. DTBSB was more active against MCF-7 and HT-29 cell lines showing remarkable IC_50_ values. However, DTBSN exhibited its highest potential against MDA-MB-231, COLO-205 and A-549 cancer cells. The IC_50_ values of both analogs were in the range of 50 to 100 μM making these compounds relevant for further lead development. Some of the IC_50_ values of DTBSB and DTBSN were comparable with the IC_50_ value of standard 5-FU. All the derivatives are active against all tested cancer cell lines.

In addition, all hybrids were virtually non-toxic to human normal peripheral blood mononuclear cells (PBMCs), indicating a high selectivity of these compounds for cancer cells. The selectivity index (SI) of each test compound for every cancer cell line was determined. The higher the SI value, the more effective and safer a drug would be in future *in vivo* experiments. The SI of a compound is a widely accepted parameter used to express a compound’s *in vitro* efficacy in the inhibition of virus replication [35]. Here, the SI values for the hybrid molecules were calculated from the ratio of their IC_50_ values in tumor cells and that of normal PBMCsl (Table 2). The selectivity index indicates the selectivity of a given compound between normal and cancer cells. The higher the magnitude of the selectivity index, the greater its tumor selectivity is [36]. The highest SI values were calculated for DTBSB including MCF-7 (8.41), MDA-MB-231 (10.61), COLO-205 (6.88), HT-29 (7.48) and A549 (8.19). In general, all the derivatives showed certain selectivity (more than 2) and the SI values entail that the test compounds were more selective towards cancer cells than the non-malignant cells.

### Molecular docking

Recently, benzopyran derivatives were investigated as potent COX-2 inhibitors and more specifically, coumarin derivatives were proved to possess potent anti-inflammatory effects and, thus, were evaluated as COX-2 inhibitors [37]. Our interest in the identification of new COX-2 inhibitors prompted us to explore the use of the coumarin framework for the design of this type of inhibitors [38]. Molecular docking was used for the exploration of the interaction between a drug and an enzyme. The determined binding energy (B. E.) values and the amino acid residues of COX-2 (PDB id: 6COX) are given in Table 3. The calculated binding energies reveal that all coumarin-quinone hybrids fit favorably into the COX-2 active site displaying hydrogen bonding with various amino acid residues of the target protein.

**Figure 5.**
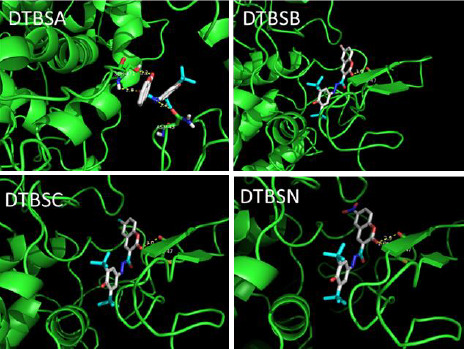
Binding of coumarin-quinone hybrids into the active site of COX-2 (6COX)

The docking ribbon structures of the COX-2 (6COX) protein with bound coumarin-quinone hybrids are given in Figure 5. The binding interactions for these hydrazones involve -NH functionalities. The best binding energy of -11.3 kcal/mol was exhibited by DTBSN followed by DTBSB, DTBSC and DTBSA. Thus, DTBSN has stronger binding interactions with COX-2 than the other derivatives. The compounds show hydrogen bonding interactions with amino acid residues and the hydrogen bond distances of 2.8 and 2.9 Å lengths indicate strong drug-enzyme interactions. These interactions lead to a stabilization of the bound compounds in the protein cavity. The docking results show that the hybrid compounds may have significant interactions with human COX-2 *in vitro*.

### ADMET and pharmacokinetic studies

ADMET Predictor is a computer program designed to predict pharmacokinetic parameters of drug-like compounds like absorption, distribution, metabolism, excretion and toxicity (ADMET) based on their molecular structures. An amenable pharmacokinetic profile with high bioactivity and low toxicities is essential for a compound, which should be investigated during drug discovery to reduce the wastage of time and resources [39-40]. The pharmacokinetic profile and drug-like properties of the new hybrid compounds were predicted using the online sources pkCSM and SwissADME to reassure the drug potential. The predicted values are shown in Table 4.

The evaluated parameters showed that the molecules have considerable solubility in water. Log S (S in mol/L) is a parameter used to evaluate aqueous solubility. All compounds have good solubility values ranging from -5.287 to -5.938 mol/L. DTBSB is more soluble in water than all other analogs. A *P*_app_ coefficient greater than 0.90 indicates that that compound has high Caco-2 permeability and is easily absorbed in the gastrointestinal tract. However, all test compounds exhibited lower *P*_app_ coefficients. DTBSN showed the highest value among all analogs, indicating a rather good absorption. Drugs with less than 30 % intestinal absorption are considered to be poorly absorbed in the intestine. All analogs were found to have good intestinal absorption, up to 94 %. With the log *K*_p_ > -2.5, the drug is considered to have relatively low skin permeability, and all analogs showed low skin permeability.

The hybrid DTBSN exhibited a distribution volume within the range (-0.15 to 0.45), which will allow a good distribution of this drug within tissues. The analog DTBSA can only cross the blood-brain barrier, while other analogs did not cross the membrane as the log BB is less than -1. The results also showed that the molecules could not inhibit CYP2D6, while all coumarin-quinone hybrids are good CYP3A4 enzyme inhibitors. This ability of the analogs indicates the ability of these compounds on the metabolism of xenobiotics in the body. The total clearance of DTBSA and DTBSN is high, while other analogs won’t be eliminated from the body. All analogs have LD_50_ values above 0.5 mM and, thus, they are non-toxic corroborating the *in vitro* results from PBMCs. The predicted results show that all analogs may cause hepatotoxicity and don’t have skin sensitization potential. These overall results of the ADMET studies disclosed that the compounds have good pharmacokinetic properties.

The physicochemical properties of the hybrid compounds were predicted using the SwissADME website. The results are presented in Table 5.

The bioavailabilities of all analogs are within the range (0.1 to 1). This profiling of the compounds brings out promising observations with respect to their pharmacological aspects.

The drug-likeness of the test compounds was established based on their physicochemical properties to find oral drug candidates. There are five different rule-based filters [41] that are in practice to determine the drug-likeness of any compound. The results of drug-likeness evaluation of the hybrid compounds are shown in Table 6

The predictions revealed that all test compounds have good drug similarity and can be suitable drug candidates for further study. They have no violation of drug-likeness according to the criteria defined by Lipinski, Ghose and Veber, but DTBSN exhibited one violation in the Egan filter due to a larger TPSA value. All analogs showed Muegge’s filter violations, corresponding to XLOGP3 > 5. According to the Five Laws, a molecule can be orally active/absorbent only if it does not violate two or more of the above rules [42]. All molecules resist Brenk’s rule due to the coumarin, quinone and imine fragments. All analogs contain a quinone moiety, which is responsible for one alert in PAINS. However, all compounds showed two violations in lead likeness due to large molecular weight (MW>350) and XLOGP3 values, which should be >3.5. Thus, these preliminary results provide the lead for the design of more potent biologically active drugs with fewer toxicities.

## Conclusions

Four new coumarin-quinone hybrids were synthesized from 3-acetylcoumarin and 2,6-di-*tert*-butyl-1,4-benzoquinone and tested for *in vitro* antimicrobial, antioxidant and anticancer activities. The results revealed that the analogs DTBSB and DTBSN were the more active antimicrobial agents when compared to the other analogs. DTBSN exhibited the best antioxidant activity and it may have a higher therapeutic potential as a chemoprotective agent against oxidative stress-related degenerative diseases. The toxicity of the synthesized hybrids was tested on human peripheral mononuclear cells (PBMCs), and they were found to be non-toxic to normal cells. The hybrids DTBSB and DTBSN exhibited good antiproliferative potential against all types of tested cancer cell lines. Compound DTBSB was more active against MCF-7 and HT-29 cells with remarkable IC_50_ values, while DTBSN exhibited its best potential against MDA-MB-231, COLO-205 and A-549 cancer cells. The IC_50_ values of both analogs are in the range of 50 to 100 μM making them suitable for further lead development. Furthermore, all derivatives showed a considerable selectivity index (more than 2), and the selectivity indexes entail that the synthesized compounds were more selective toward cancer cells than the non-malignant cells. It is appropriate to mention that antibiotics interfere with the normal functioning of living cells [43]. This property is useful for controlling the growth of microorganisms, but at the same time, it can act as a double-edged sword and can also interfere with the functioning of metabolically active, rapidly multiplying cancer cells [44]. The compounds in the present work have shown activity against the cancer cell lines as well as against microorganisms. The observed antioxidant property of these compounds enables them to reduce oxidative stress, play chemoprotective agents' role in the biological system, and protect cells from oxidative stress-induced neoplastic changes. The *in vitro* results appear to be in agreement with the observations of the molecular docking study. The pharmacokinetics and drug-likeness evaluation showed a high level of GI absorption of up to 94 %, while all analogs have good solubility in water. The *V*_Dss_, BBB membrane permeability (log BB) and CNS permeability were used to characterize the distribution of the new compounds and all analogs have a fair distribution in the body. All analogs did not inhibit CYP2D6 and but they were good CYP3A4 inhibitors depicting the ability of these analogs to metabolize xenobiotics in the body. The predictions also underlined the non-toxic nature of these analogs, which is reflected through high values of total clearance, non-hepatotoxic nature and no skin sensitization. These results of ADMET studies revealed that the compounds have acceptable ADMET and pharmacokinetic properties. Furthermore, the drug-likeness study revealed that most of the compounds fulfilled all requirements of Lipinski, Ghose and Veber rules, only DTBSN exhibited one violation in the Egan filter due to a larger TPSA value. All analogs showed Muegge’s filter violations. The compounds possess a quinone moiety leading to one alert in PAINS. Further *in vitro* and *in vivo* studies will be necessary for the confirmation of the chemoinformatics investigation. These preliminary results may help get a lead structure with selective anticancer potential.



## Figures and Tables

**Table 1.** IC_50_ concentration values of coumarin-quinone hybrids

Microorganism	*Staphylococcus aureus*	*Pseudomonas aeruginosa*	*Candida albicans*
**Compound**	IC_50_ Drug Concentrations (μM)		
DTBSA	236.42 ± 2.90	225.26 ± 3.08	260.19 ± 0.78
DTBSC	250.04 ± 3.37	275.70 ± 0.77	246.19 ± 8.18
DTBSB	107.28 ± 1.23	129.63 ± 2.50	121.04 ± 1.10
DTBSN	134.67 ± 2.94	172.59 ± 2.08	163.75 ± 2.58
STR	76.96 ± 4.46	93.14 ± 7.64	--
ITR	--	--	56.49 ± 0.82

**Table 2.** Calculated IC_50_ values (μM) and Selectivity Index (SI) of coumarin-quinone hybrids

Cell Lines	MCF-7	MDA-MB-231	COLO-205	HT-29	A-549	PBMC
**Compounds**	IC_50_ Drug Concentrations					
DTBSA	120.5 ± 2.54	69.18 ± 6.45	109.99 ± 2.93	112.33 ± 1.58	103.30 ± 0.17	429.85 ± 3.63
DTBSC	107.88 ± 2.47	58.81 ± 7.38	108.93 ± 0.76	107.02 ± 2.43	88.50 ± 2.01	423.97 ± 2.06
DTBSB	63.93 ± 2.61	50.68 ± 4.31	78.18 ± 0.45	71.82 ± 3.61	65.62 ± 4.33	537.51 ± 5.37
DTBSN	76.84 ± 4.17	53.29 ± 2.22	78.19 ± 2.81	100.30 ± 1.97	66.12 ± 0.04	492.3 ± 4.75
5-FU	39.72 ± 1.68	46.76 ± 8.59	62.23 ± 2.04	56.46 ± 2.64	48.15 ± 1.56	---
	Selectivity Index (SI)					
DTBSA	3.57	6.21	3.91	3.83	4.16	---
DTBSC	3.93	7.21	3.89	3.96	4.79	---
DTBSB	8.41	10.61	6.88	7.48	8.19	---
DTBSN	6.41	9.24	6.30	4.91	7.45	---

**Table 3.** Binding energies and the interacting surfaces of the compounds

No.	Code	B.E. in kcal/mol	Amino acid Residue	Bond Length in Å
**1**	DTBSA	-8.7	SER-471, ASN-43	3.2, 2.8, 3.4
**2**	DTBSC	-11.1	CYS-47	3.0
**3**	DTBSB	-11.1	CYS-47	3.0
**4**	DTBSN	-11.3	CYS-47	2.8, 2.9

**Table 4.** ADMET properties of coumarin-quinone hybrids calculated with pkCSM online tool

Property	Model Name	DTBSA	DTBSC	DTBSB	DTBSN
**Absorption**	Water solubility^a^	-5.287	-5.864	-5.938	-5.373
	Caco-2 permeability^b^	0.597	0.611	0.602	0.632
	Intestinal absorption (human)^c^	93.878	93.183	93.116	94.408
	Skin permeability^d^	-2.877	-2.876	-2.873	-2.754
**Distribution**	VDss (human)^e^	-0.01	-0.031	-0.021	-0.188
	BBB permeability^f^	0.139	-0.015	-0.024	-0.227
	CNS permeability^g^	-1.665	-1.553	-1.531	-1.888
**Metabolism**	CYP2D6 inhibitor^h^	No	No	No	No
	CYP3A4 inhibitor^h^	Yes	Yes	Yes	Yes
**Excretion**	Total clearance^i^	0.598	-0.373	-0.395	0.57
**Toxicity**	Oral rat acute toxicity^j^	2.896	3.035	3.04	2.797
	Oral rat chronic toxicity^k^	1.367	1.239	1.211	1.812
	Hepatotoxicity^h^	Yes	Yes	Yes	Yes
	Skin sensitization^h^	No	No	No	No

^a^(log mol/L)

^b^(log Papp in 10-6 cm/s)

^c^(% Absorbed)

^d^(log Kp)

^e^(log L/kg)

^f^(Fu)

^g^(log PS)

^h^(Yes/No)

^i^(log ml/min/kg)

^j^(LD_50_ in mol/kg)

^k^(LOAEL in log mg/kg_bw/day)

**Table 5.** Physicochemical properties of the new coumarin-quinone hybrids

Properties		DTBSA	DTBSC	DTBSB	DTBSN
Molecular weight (g/mol)		406.47	440.92	485.37	451.47
No. of heavy atoms		30	31	31	33
No. of arom. heavy atoms		10	10	10	10
No. of rotatable bonds		5	5	5	6
No. of H-Bond acceptors		5	5	5	7
No. of H-Bond donors		1	1	1	1
Molar refractivity		118.27	123.28	125.97	127.10
Total polar surface area Å^2^		88.74	88.74	88.74	134.56
Solubility	log S (ESOL)	-5.80	-6.40	-6.71	-5.88
	log S (Ali)	-7.22	-7.87	-7.93	-8.00
	log S (SILICOS-IT)	-7.04	-7.62	-7.81	-6.38
Lipophilicity	MLOGP	3.11	3.59	3.69	2.23
	WLOGP	4.41	5.06	5.17	4.31
	XLOGP3	5.59	6.22	6.28	5.42

**Table 6.** Drug likeness evaluation of the new coumarin-quinone hybrids

Rule-based filters	DTBSA	DTBSC	DTBSB	DTBSN
Lipinski violations	0	0	0	0
Ghose violations	0	0	0	0
Veber violations	0	0	0	0
Egan violations	0	0	0	1 (TPSA > 131.6)
Muegge violations	1 (XLOGP3 >5)	1 (XLOGP3 >5)	1 (XLOGP3 >5)	1 (XLOGP3 >5)
Bioavailability score	0.55	0.55	0.55	0.55
PAINS No. of alerts	1 (Quinone)	1 (Quinone)	1 (Quinone)	1 (Quinone)
Brenk No. of alerts	3 (quinone, coumarin and imine)	3 (quinone, coumarin and imine)	3 (quinone, coumarin and imine)	5 (quinone, coumarin, imine, nitro group and N-O bond)
Lead likeness No. of violations	2 (MW>350, XLOGP3>3.5)	2 (MW>350, XLOGP3>3.5)	2 (MW>350, XLOGP3>3.5)	2 (MW>350, XLOGP3>3.5)

## References

[1] Nofal Z.M. El-Zahar M. I. Abd El-Karim S.S. Novel coumarin derivatives with expected biological activity. Molecules 5(2) (2000) 99-113. https://doi.org/10.3390/50200099. 10.3390/50200099

[2] Pangal A. Mujahid Y. Desai B. Shaikh J.A. Ahmed K. Synthesis of 3-(2-(subsituted-(trifluoromethyl)phenylamino)acetyl)-2H-chromen-2-one derivatives as new anticancer agents. Current Chemistry Letters 11(1) (2022) 105–112. https://doi.org/10.5267/j.ccl.2021.8.004. 10.5267/j.ccl.2021.8.004

[3] Wu Y. Xu J. Liu Y. Zeng Y. Wu G. A review on antitumor mechanisms of coumarins. Front. Oncol. 10 (2020) 592853. https://doi.org/10.3389/fonc.2020.592853. 10.3389/fonc.2020.592853 33344242 PMC7746827

[4] Song X.F. Fan J. Liu L. Liu X.F. Gao F. Coumarin derivatives with anticancer activities: An update. Arch. Pharm. (Weinheim) 353 (2020) e2000025. https://doi.org/10.1002/ardp.202000025 10.1002/ardp.202000025 32383190

[5] Kontogiorgis C. Hadjipavlou-Litina D. Biological evaluation of several coumarin derivatives designed as possible anti-inflammatory/antioxidant agents. J Enzyme Inhib. Med. Chem. 18 (2003) 63-69. https://doi.org/10.1080/1475636031000069291 10.1080/1475636031000069291 12751823

[6] Feng D. Zhang A. Yang Y. Yang P. Coumarin-containing hybrids and their antibacterial activities. Archiv der Pharmazie 353(6) (2020) e1900380. https://doi.org/10.1002/ardp.201900380 10.1002/ardp.201900380 32253782

[7] Al-Majedy Y.K. Kadhum A.A.H. Al-Amiery A.A. Mohamad A.B. Coumarins: The antimicrobial agents. Sys. Rev. Pharm. 8(1) (2017) 24-30. http://dx.doi.org/10.5530/srp.2017.1.11 10.5530/srp.2017.1.11

[8] Wei Y. Peng W. Wang D. Hao S.H. Li W. W. Ding F. Design, synthesis, antifungal activity, and 3D-QSAR of coumarin derivatives. J. Pestic. Sci. 43 (2018) 88-95. https://doi.org/10.1584%2Fjpestics.D17-075 10.1584/2Fjpestics.D17-075 30363100 PMC6140650

[9] Xu Z. Chen Q. Zhang Y. Liang C. Coumarin-based derivatives with potential anti-HIV activity. Fitoterapia 150 (2021) 104863. https://doi.org/10.1016/j.fitote.2021.104863 10.1016/j.fitote.2021.104863 33582266

[10] Dai H. Huang M. Qian J. Liu J. Meng C. Li Y. Ming G. Zhang T. Wang S. Shi Y. Yao Y. Ge S. Zhang Y. Ling Y. Excellent antitumor and antimetastatic activities based on novel coumarin/pyrazole oxime hybrids. Eur. J. Med. Chem. 166 (2019) 470-479. https://doi.org/10.1016/j.ejmech.2019.01.070 10.1016/j.ejmech.2019.01.070 30739827

[11] Bhattarai N. Kumbhar A.A. Pokharel Y.R. Yadav P. N. Anticancer potential of coumarin and its derivatives. Mini Rev. Med. Chem. 21 (2021) 2996-3029. https://doi.org/10.2174/1389557521666210405160323 10.2174/1389557521666210405160323 33820507

[12] Finn P.R. Motivation, working memory, and decision making: a cognitive-motivational theory of personality vulnerability to alcoholism. Behav. Cogn. Neurosci. Rev. 1 (2002) 183-205. https://doi.org/10.1177/1534582302001003001 10.1177/1534582302001003001 17715592

[13] Behzadi S.A. Sheikhhosseini E. Ahmadi S.A. Ghazanfari D. Akhgar M. Synthesis and characterization of novel biological tetracoumarin derivatives bearing ether moieties. Heterocyclic Comm. 26(1) (2020) 60-67. https://doi.org/10.1515/hc-2020-0009 10.1515/hc-2020-0009

[14] Asche C. Antitumour quinones. Mini Rev. Med. Chem. 5 (2005) 449-467. https://doi.org/10.2174/1389557053765556 10.2174/1389557053765556 15892687

[15] Aly A.A. Hassan A.A. Mohamed N.K. Ramadan M. Abd El-Aal A.S. Braese S. Nieger M. Synthesis of quinone-based heterocycles of broad-spectrum anticancer activity. J. Chem. Res. 45(5-6) (2021) 562-571. https://doi.org/10.1177/1747519820959737 10.1177/1747519820959737

[16] Shaterzadeh-Yazdi H. Noorbakhsh M.F. Hayati F. Samarghandian S. Farkhondeh T. Immunomodulatory and Anti-inflammatory Effects of Thymoquinone. Cardiovasc Hematol Disord Drug Targets 18 (2018) 52-60. https://doi.org/10.2174/1871529x18666180212114816 10.2174/1871529x18666180212114816 29437018

[17] Wellington K.W. Kolesnikova N.I. Nyoka N.B.P. McGaw L.J. Investigation of the antimicrobial and anticancer activity of aminonaphthoquinones. Drug Dev. Re.s 80 (2019) 138-146. https://doi.org/10.1002/ddr.21477 10.1002/ddr.21477 30284739

[18] Wellington K.W. Understanding cancer and anticancer activities of naphthoquinones-a review. RSC Adv. 5 (2015) 20309-20338. https://doi.org/10.1039/C4RA13547D 10.1039/C4RA13547D

[19] Bhasin D. Chettiar S.N. Etter J.P. Mok M. Li P.K. Anticancer activity and SAR studies of substituted 1,4-naphthoquinones. Bioorg. Med. Chem. 21 (2013) 4662-4669. https://doi.org/10.1016%2Fj.bmc.2013.05.017 10.1016/2Fj.bmc.2013.05.017 23791367 PMC4304211

[20] Kadela-Tomanek M. Bębenek E. Chrobak E. Latocha M. Boryczka S. Alkoxy and Enediyne Derivatives Containing 1,4-Benzoquinone Subunits-Synthesis and Antitumor Activity. Molecules 22 (2017) E447. https://doi.org/10.3390%2Fmolecules22030447 10.3390/2Fmolecules22030447 PMC6155387 28287461

[21] Abraham I. Joshi R. Pardasani P. Pardasani R.T. Recent advances in 1,4-benzoquinone chemistry. J. Braz. Chem. Soc. 22(3) 2011 385-421. https://doi.org/10.1590/S0103-50532011000300002 10.1590/S0103-50532011000300002

[22] Gupta S.P. Quantitative structure-activity relationship studies on anticancer drugs. Chem. Rev. 94 (1994) 1507–1551. https://doi.org/10.1021/cr00030a003 10.1021/cr00030a003

[23] da Silva A.J. M. Netto C.D. Pacienza-Lima W. Torres-Santos E.C. Rossi-Bergmann B. Maurel S. Valentind A. Costa P.R.R. Antitumoral, antileishmanial and antimalarial activity of pentacyclic 1,4-naphthoquinone derivatives. J. Braz. Chem. Soc. 20(1) (2009) 176-182. http://dx.doi.org/10.1590/S0103-50532009000100026 10.1590/S0103-50532009000100026

[24] Amaro A.R. Oakley G.G. Bauer U. Spielmann H.P. Robertson L.W. Metabolic activation of PCBs to quinones: reactivity toward nitrogen and sulfur nucleophiles and influence of superoxide dismutase. Chem. Re.s Toxicol. 9 (1996) 623-629. https://doi.org/10.1021/tx950117e 10.1021/tx950117e 8728508

[25] O'Brien P.J. Molecular mechanisms of quinone cytotoxicity. Chem. Biol. Interact. 80 (1991) 1-41. https://doi.org/10.1016/0009-2797(91)90029-7 10.1016/0009-2797(91)90029-7 1913977

[26] Lin T.S. Zhu L.Y. Xu S.P. Divo A.A. Sartorelli A.C. Synthesis and antimalarial activity of 2-aziridinyl- and 2,3-bis(aziridinyl)-1,4-naphthoquinonyl sulfonate and acylate derivatives. J. Med. Chem. 34 (1991) 1634-1639. https://doi.org/10.1021/jm00109a016 10.1021/jm00109a016 2033589

[27] Lin A.J. Lillis B.J. Sartorelli A.C. Potential bioreductive alkylating agents. 5. Antineoplastic activity of quinoline-5,8-diones, naphthazarins, and naphthoquinones. J. Med. Chem. 18 (1975) 917-921. https://doi.org/10.1021/jm00243a012 10.1021/jm00243a012 1159713

[28] Deniz N.G. Ibis C. Gokmen Z. Stasevych M. Novikov V. Komarovska-Porokhnyavets O. Ozyurek M. Guclu K. Karakas D. Ulukaya E. Design, synthesis, biological evaluation, and antioxidant and cytotoxic activity of heteroatom-substituted 1,4-naphtho- and benzoquinones. Chem. Pharm. Bull. (Tokyo) 63(12) (2015) 1029-1039. https://doi.org/10.1248/cpb.c15-00607 10.1248/cpb.c15-00607 26633024

[29] Isyaku Y. Uzairu A. Uba S. Computational studies of a series of 2-substituted phenyl-2-oxo-, 2-hydroxyl- and 2-acylloxyethylsulfonamides as potent antifungal agents. Heliyon 6 (2020) e03724. https://doi.org/10.1016/j.heliyon.2020.e03724 10.1016/j.heliyon.2020.e03724 32322718 PMC7160569

[30] Nisha C.M. Kumar A. Nair P. Gupta N. Silakari C. Tripathi T. Kumar A. Molecular docking and *in silico* ADMET study reveals acylguanidine 7a as a potential inhibitor of β-secretase. Adv. Bioinformatics 2016 (2016) 9258578. https://doi.org/10.1155/2016/9258578 10.1155/2016/9258578 27190510 PMC4842033

[31] Singh D.B. Gupta M.K. Singh D.V. Singh S.K. Misra K. Docking and *in silico* ADMET studies of noraristeromycin, curcumin and its derivatives with *Plasmodium falciparum* SAH hydrolase: a molecular drug target against malaria. Interdiscip. Sci. 5 (2013) 1-12. https://doi.org/10.1007/s12539-013-0147-z 10.1007/s12539-013-0147-z 23605635

[32] Żołek T. Maciejewska D. Theoretical evaluation of ADMET properties for coumarin derivatives as compounds with therapeutic potential. Eur. J. Pharm. Sci. 109 (2017) 486-502. https://doi.org/10.1016/j.ejps.2017.08.036 10.1016/j.ejps.2017.08.036 28867490

[33] Jenny M. Klieber M. Zaknun D. Schroecksnadel S. Kurz K. Ledochowski M. Schennach H. Fuchs D. *In vitro* testing for anti-inflammatory properties of compounds employing peripheral blood mononuclear cells freshly isolated from healthy donors. Inflamm. Res. 60 (2011) 127-135. https://dx.doi.org/10.1007/s00011-010-0244-y 10.1007/s00011-010-0244-y 20740299

[34] Trott O. Olson A.J. AutoDock Vina: improving the speed and accuracy of docking with a new scoring function, efficient optimization, and multithreading. J. Comput. Chem. 31 (2010) 455-461. https://doi.org/10.1002%2Fjcc.21334 10.1002/2Fjcc.21334 19499576 PMC3041641

[35] Hashemi Goradel N. Najafi M. Salehi E. Farhood B. Mortezaee K. Cyclooxygenase-2 in cancer: A review. J. Cell Physiol. 234 (2019) 5683-5699. https://doi.org/10.1002/jcp.27411 10.1002/jcp.27411 30341914

[36] Singh K. Gangrade A. Jana A. Mandal B.B. Das N. Design, synthesis, characterization, and antiproliferative activity of organoplatinum compounds bearing a 1,2,3-triazole ring. ACS Omega 4 (2019) 835–841. https://doi.org/10.1021/acsomega.8b02849 10.1021/acsomega.8b02849

[37] Badmus J.A. Ekpo O.E. Hussein A.A. Meyer M. Hiss D.C. Cytotoxic and cell cycle arrest properties of two steroidal alkaloids isolated from *Holarrhena floribunda* (G. Don) T. Durand & Schinz leaves. BMC Complement. Altern. Med. 19 (2019) 112. https://doi.org/10.1186/s12906-019-2521-9 10.1186/s12906-019-2521-9 31151442 PMC6545003

[38] Dawood D.H. Batran R.Z. Farghaly T.A. Khedr M.A. Abdulla M.M. New coumarin derivatives as potent selective cox-2 inhibitors: synthesis, anti-inflammatory, QSAR, and molecular modeling studies. Arch. Pharm. (Weinheim) 348 (2015) 875-888. https://doi.org/10.1002/ardp.201500274 10.1002/ardp.201500274 26462142

[39] Aqlan F.M. Synthesis, ADMET and docking studies of novel pyrazoles incorporating coumarin moiety as tyrosine kinase (Src) inhibitors. Biointerface Res. Appl. Chem. 11(5) (2021) 13706-13714. https://doi.org/10.33263/BRIAC115.1370613714 10.33263/BRIAC115.1370613714

[40] Han Y. Zhang J. Hu C.Q. Zhang X. Ma B. Zhang P. *In silico* ADME and toxicity prediction of ceftazidime and its impurities. Front. Pharmacol. 10 (2019) 434. https://doi.org/10.3389/fphar.2019.00434 10.3389/fphar.2019.00434 31068821 PMC6491819

[41] Daina A. Zoete V. A BOILED-Egg to predict gastrointestinal absorption and brain penetration of small molecules. Chem. Med. Chem. 11 (2016) 1117-1121. https://doi.org/10.1002/cmdc.201600182 10.1002/cmdc.201600182 27218427 PMC5089604

[42] Bickerton G.R. Paolini G.V. Besnard J. Muresan S. Hopkins A.L. Quantifying the chemical beauty of drugs. Nat. Chem. 4 (2012) 90-98. https://doi.org/10.1038%2Fnchem.1243 10.1038/2Fnchem.1243 22270643 PMC3524573

[43] Barrios-González J. Mejýa A. Production of Antibiotics and other Commercially Valuable Secondary Metabolites. In: Current Developments in Solid-state Fermentation Pandey A. Soccol C. R. Larroche C. (eds)., Springer, New York, NY, USA, 2008, 302–336. https://doi.org/10.1007/978-0-387-75213-6_14 10.1007/978-0-387-75213-6_14

[44] Xia D. Yang X. Liu W. Shen F. Pan J. Lin Y. Du N. Sun Y. Xi X. Over-expression of CHAF1A in Epithelial Ovarian Cancer can promote cell proliferation and inhibit cell apoptosis. Biochem. Biophys. Res. Commun. 486(1) (2017) 191-197. https://doi.org/10.1016/j.bbrc.2017.03.026 10.1016/j.bbrc.2017.03.026 28286267

