# Supplementary material for: Synthesis and biological evaluation of coumarin-quinone hybrids as multifunctional bioactive agents
Source: ADMET DMPK. 2022 Oct 7;11(1):81–96. doi: 10.5599/admet.1468 (PMC9909729; doi:10.5599/admet.1468)
Supplement: Supplementary file 1 [file ADMET-11-1468-S1.pdf]

doi: https://doi.org/10.5599/admet.1468

Open Access : ISSN : 1848-7718
http://www.pub.iapchem.org/ojs/index.php/admet/index

Supplementary data

Synthesis and biological evaluation of coumarin-quinone hybrids as multifunctional bioactive agents

Anees Pangal[1,2] and Khursheed Ahmed[1,2]*

[1] *Dept. of Chemistry & Post Graduate Centre, Abeda Inamdar Sr. College of Arts, Science & Commerce (Autonomous), Camp, Pune – 411001, INDIA*
[2] *Advanced Scientific Research Laboratory, Azam Campus, Pune – 411001, India.*

*Corresponding Author: E-mail: khursheedahmed@azamcampus.org

Received: MMMM DD, YYYY; Revised: MMMM DD, YYYY; Published: MMMM DD, YYYY

Supplementary document

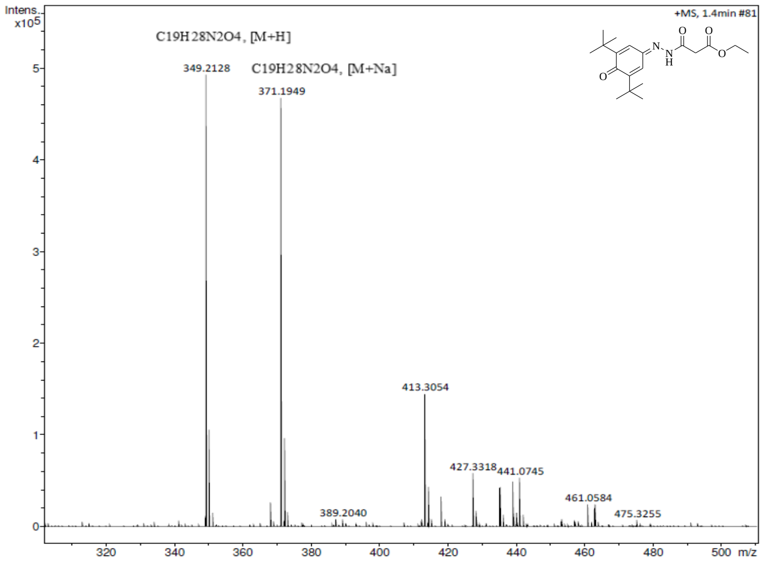

Mass Spectrum of DTBH

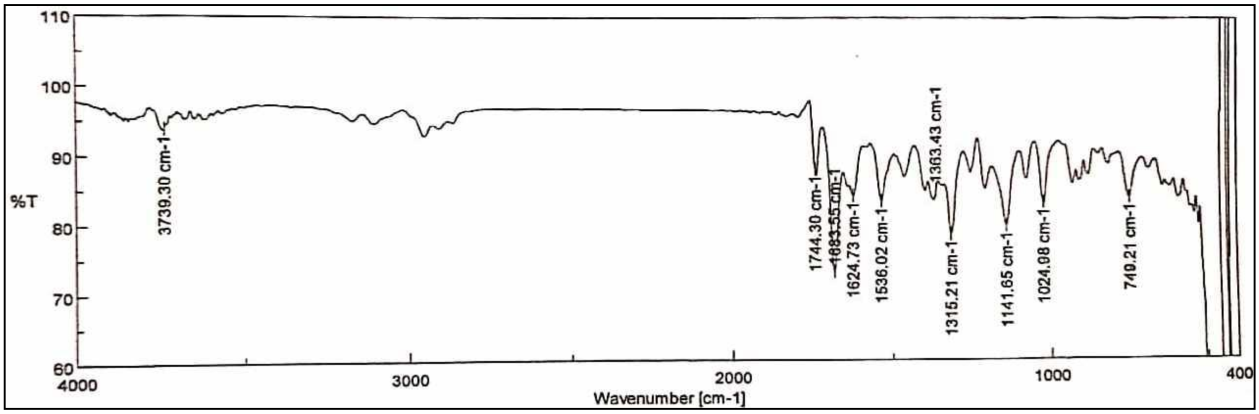

Infra Red Spectrum of DTBH

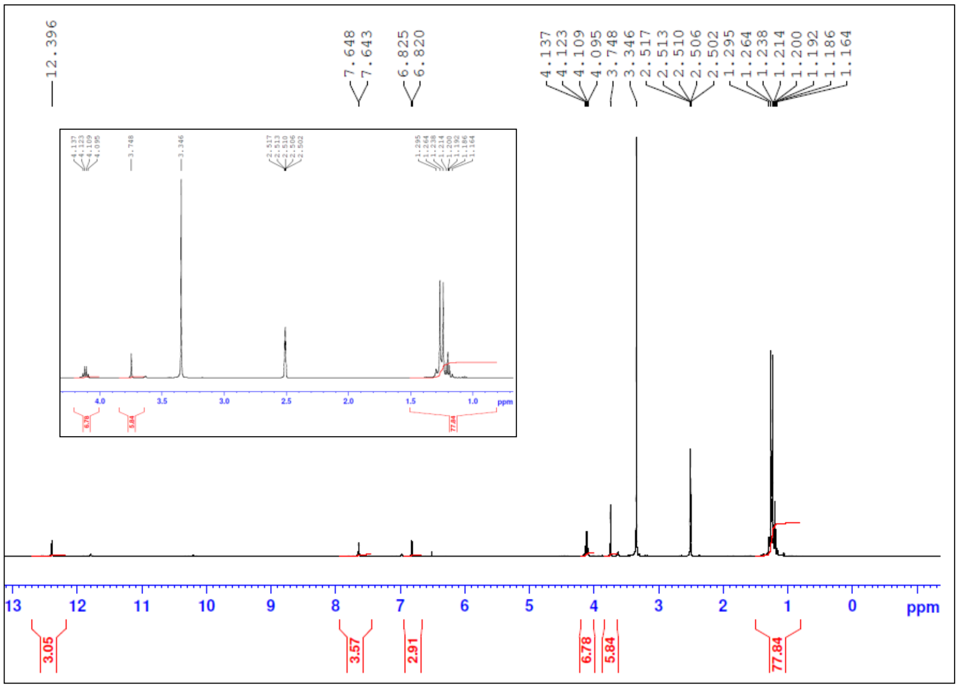

^{1}H-NMR Spectrum of DTBH

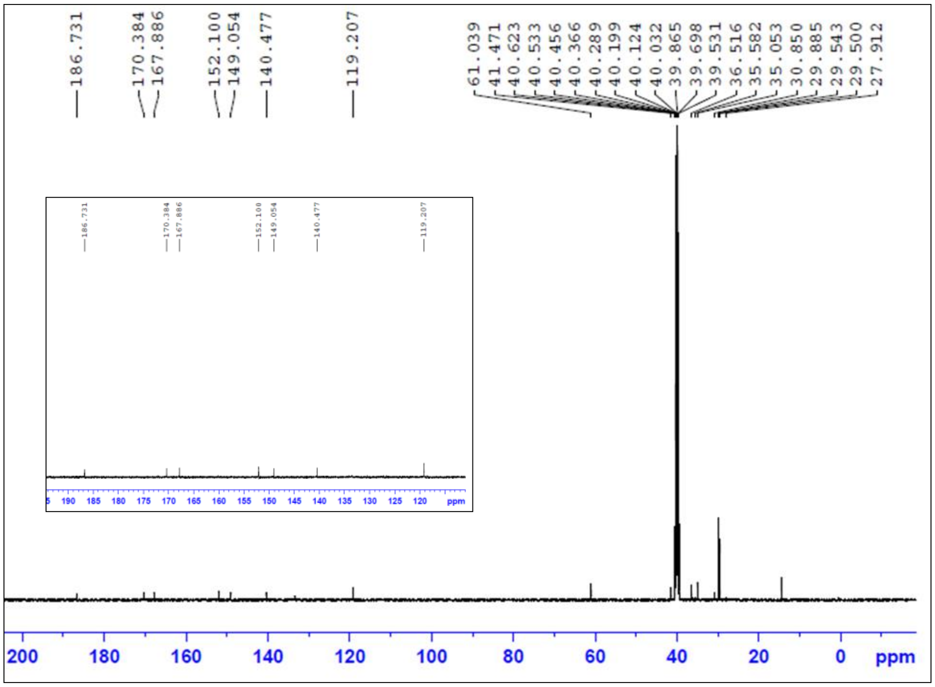

^{13}C-NMR Spectrum of DTBH

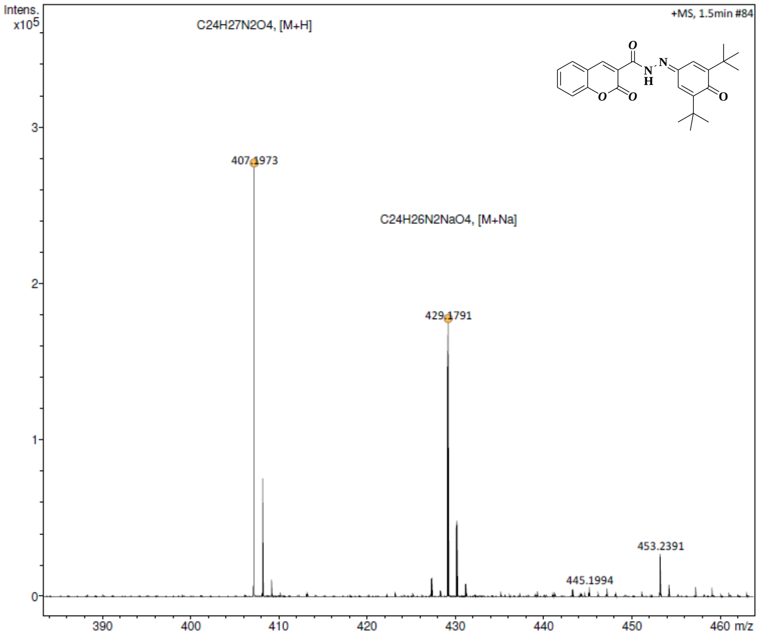

Mass Spectrum of DTBSA

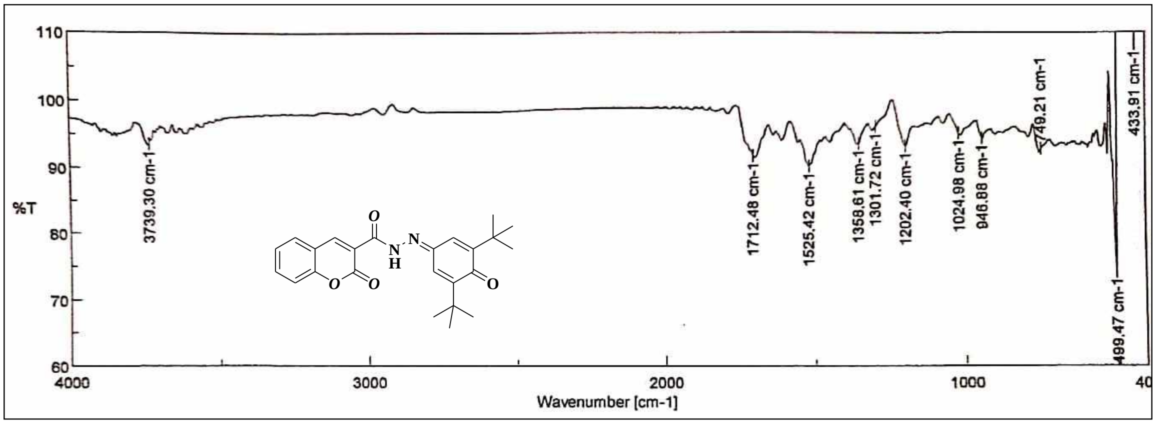

Infra Red Spectrum of DTBSA

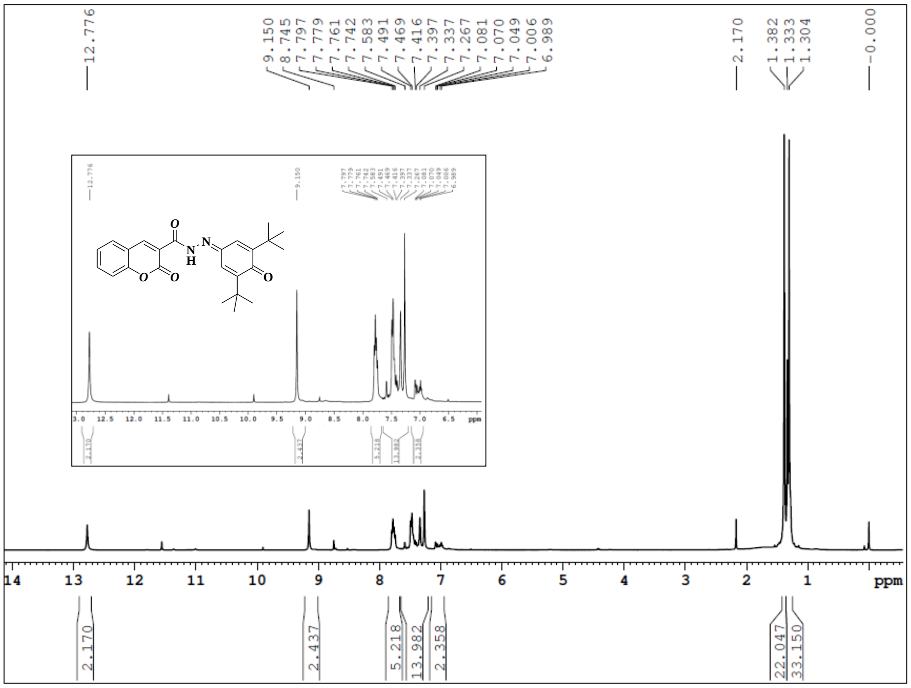

^{1}H-NMR Spectrum of DTBSA

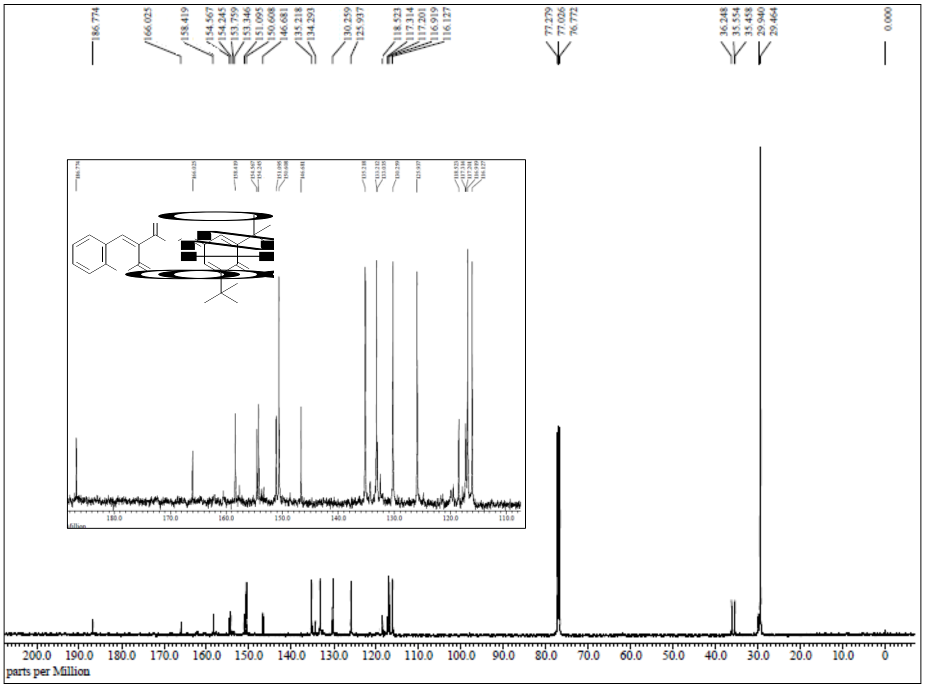

^{13}C-NMR Spectrum of DTBSA

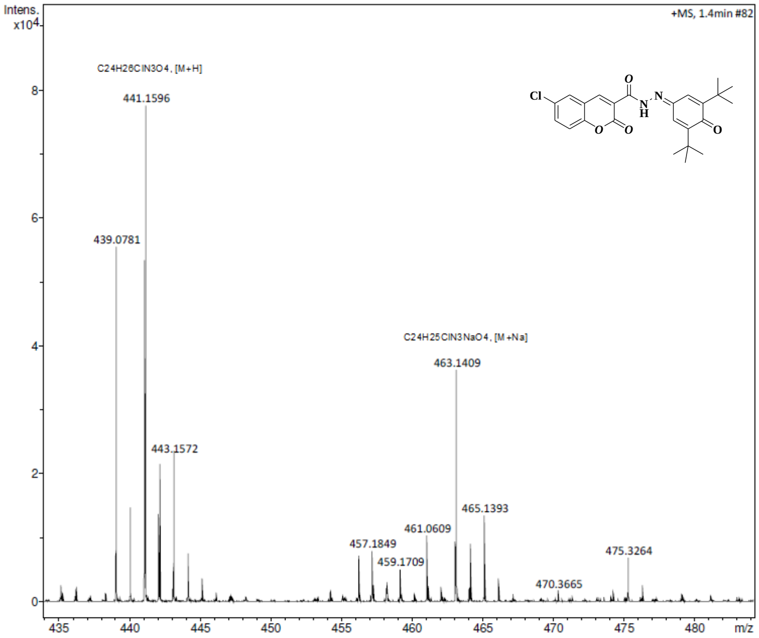

Mass Spectrum of DTBSC

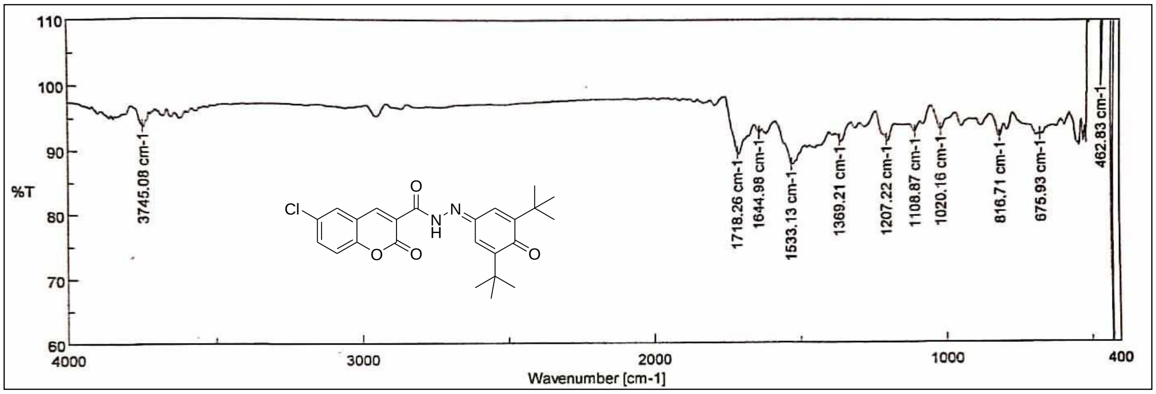

Infra Red Spectrum of DTBSC

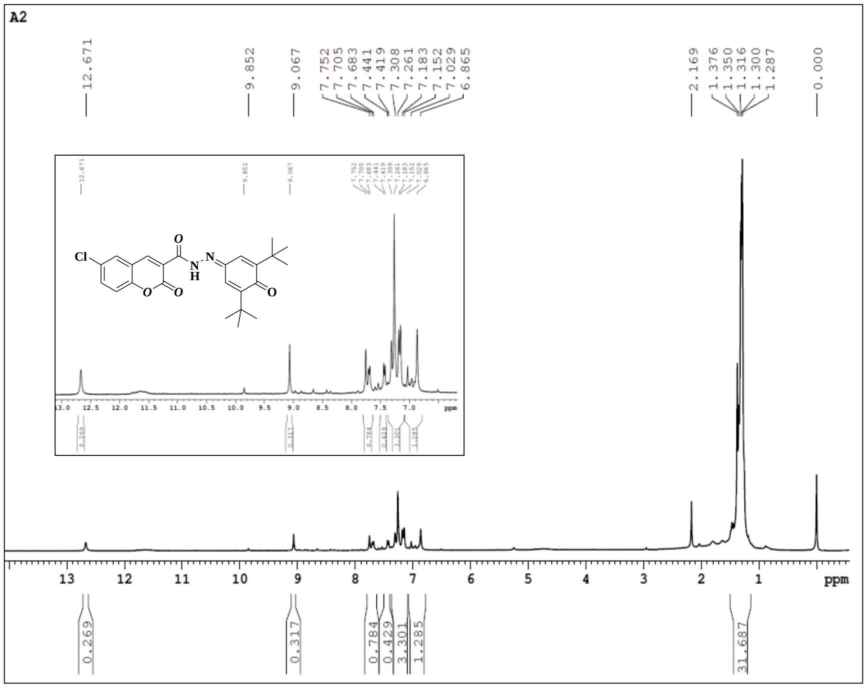

^{1}H-NMR Spectrum of DTBSC

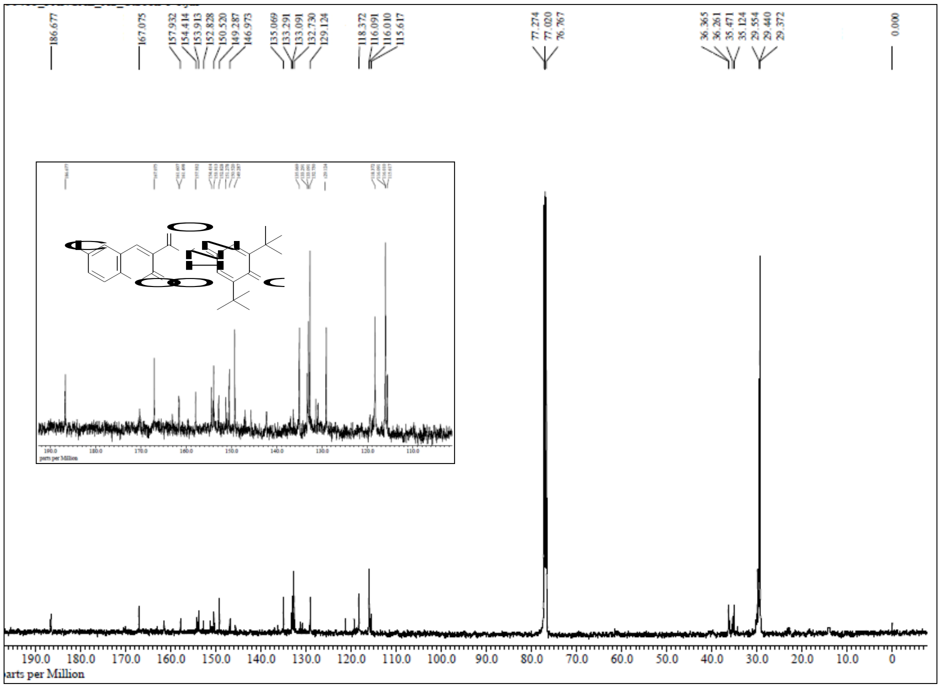

^{13}C-NMR Spectrum of DTBSC

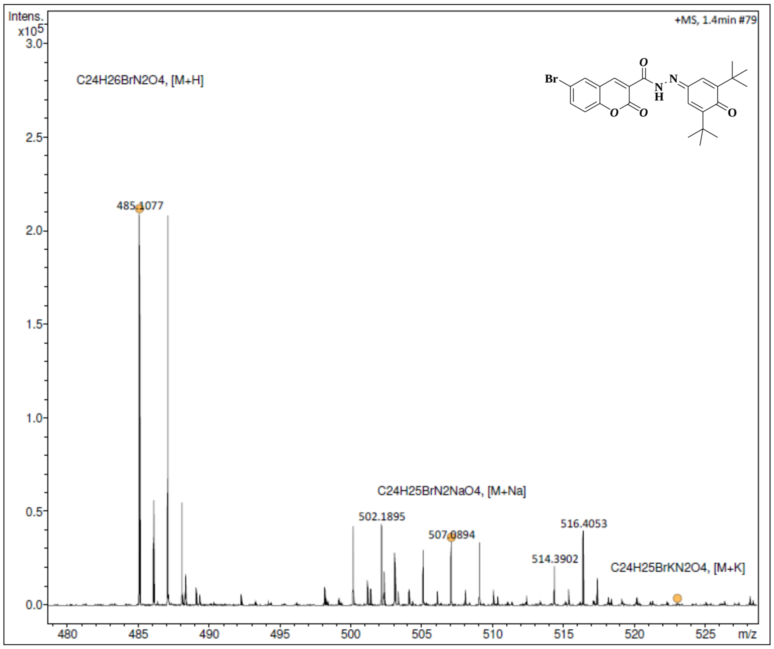

Mass Spectrum of DTBSB

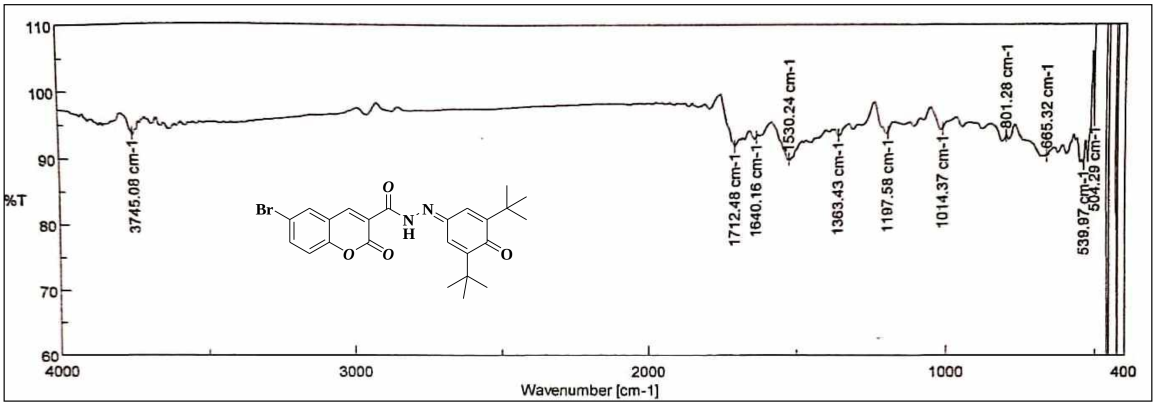

Infra Red Spectrum of DTBSB

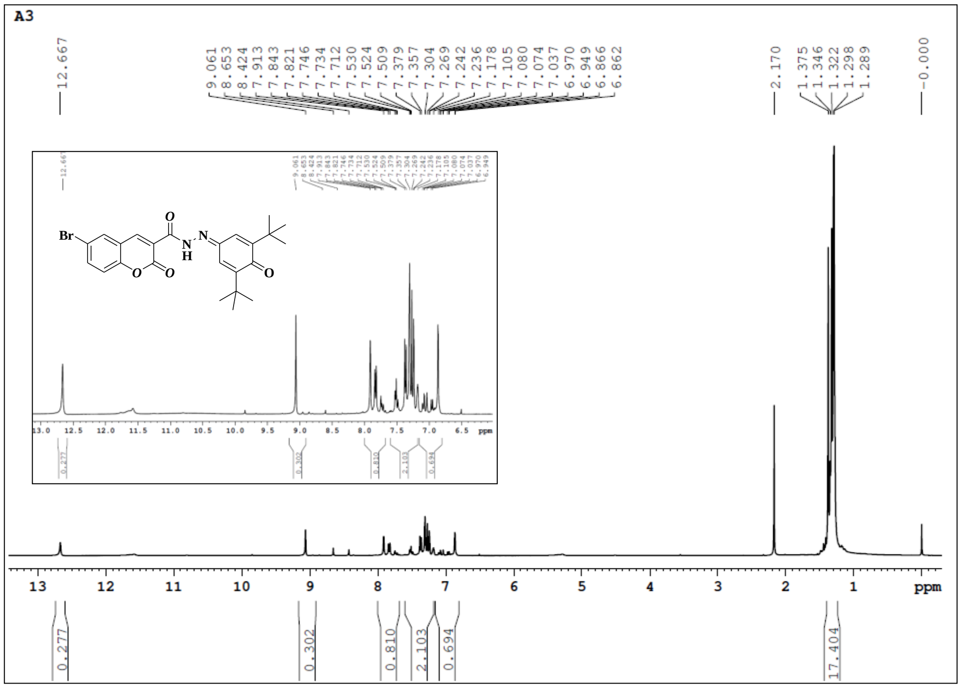

^{1}H-NMR Spectrum of DTBSB

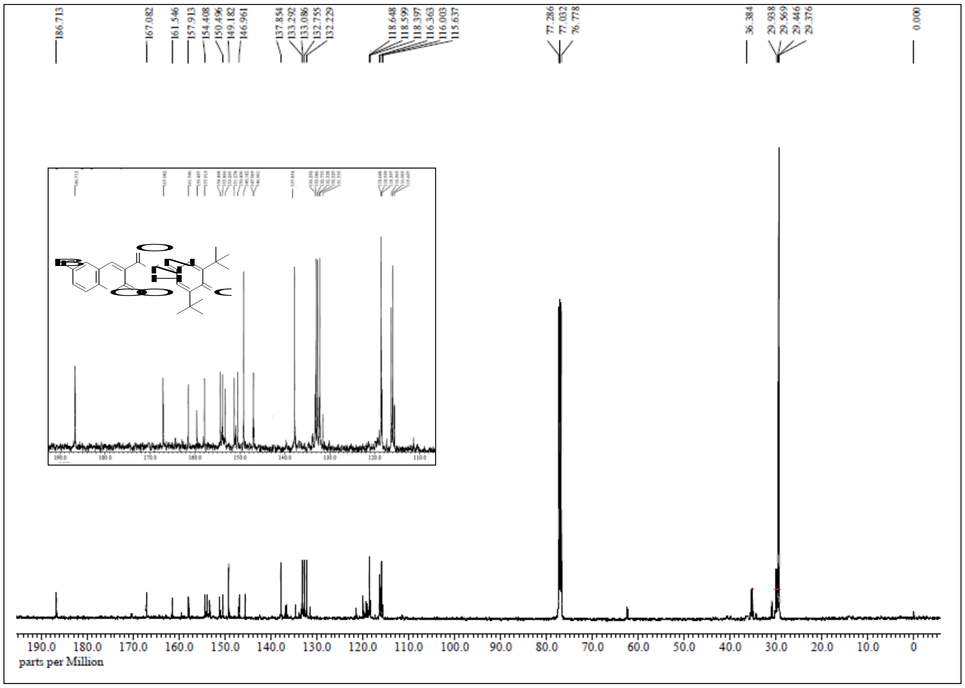

^{13}C-NMR Spectrum of DTBSB

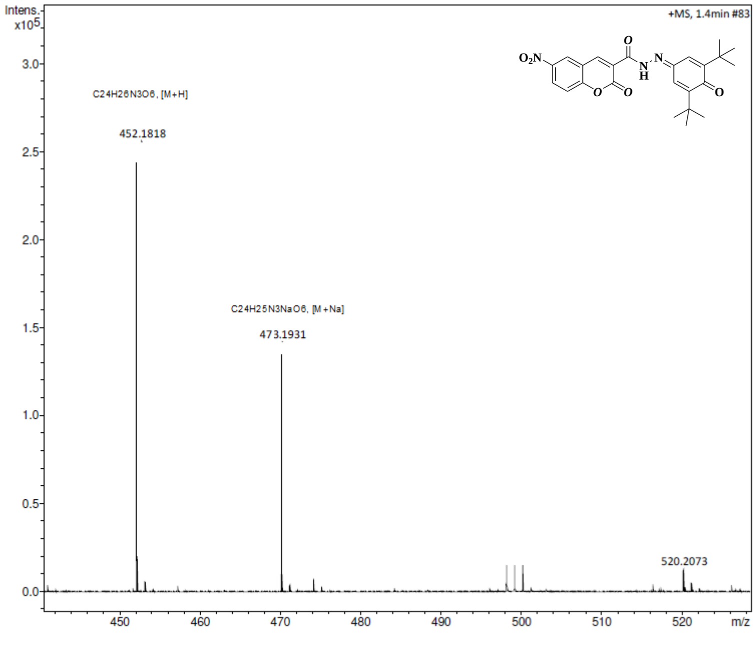

Mass Spectrum of 3ACDT

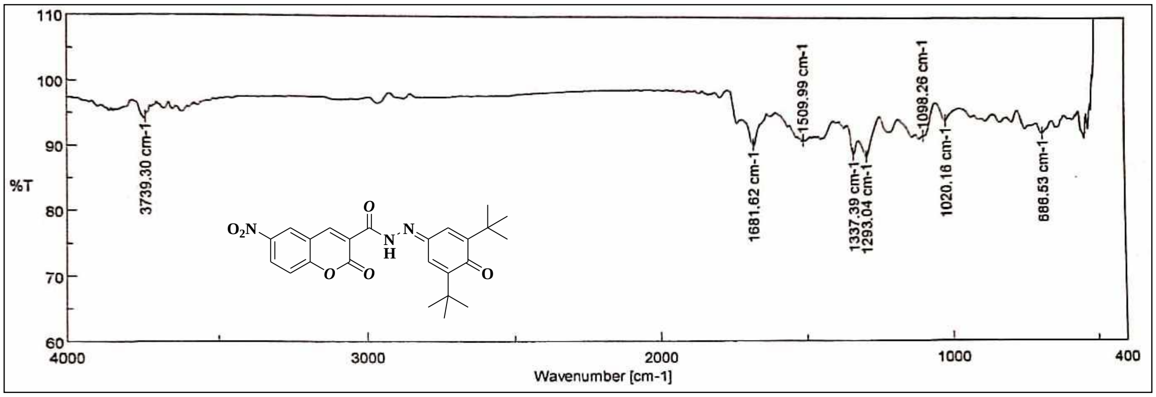

Infra Red Spectrum of DTBSN

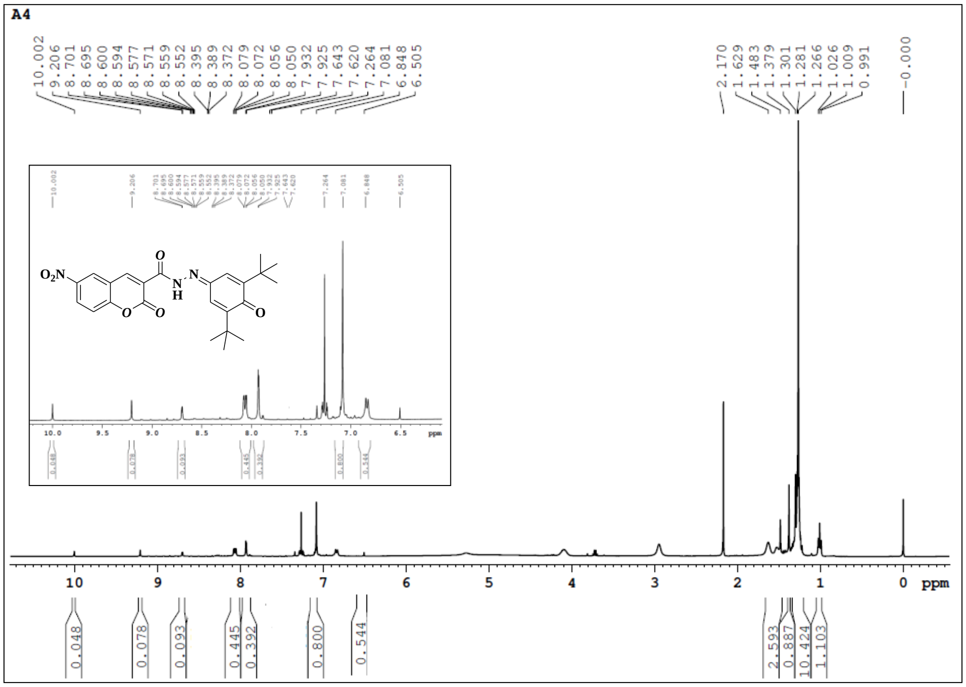

^{1}H-NMR Spectrum of DTBSN

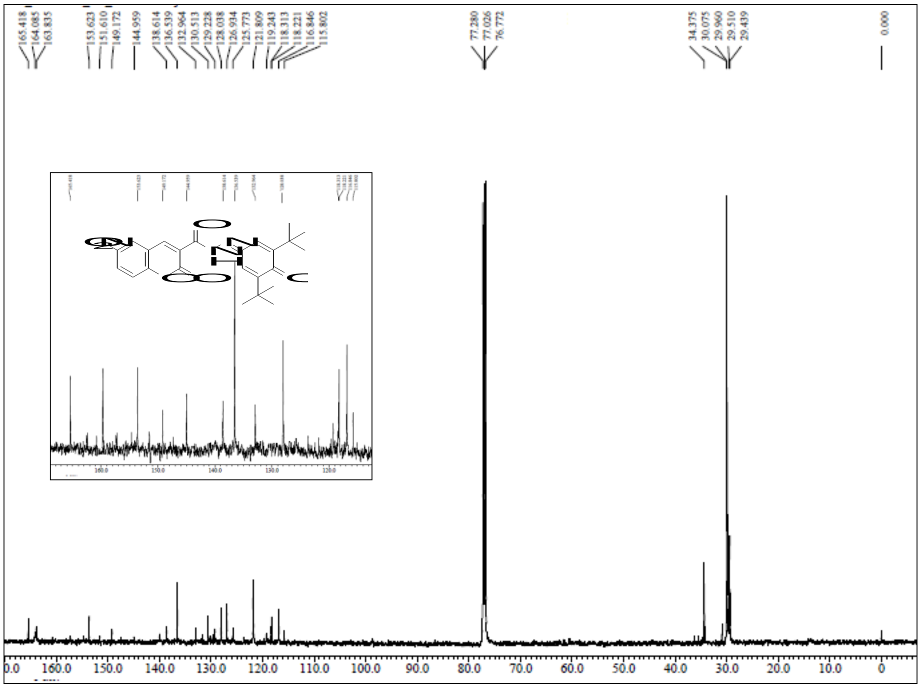

^{13}C-NMR Spectrum of DTBSN
